# Exposure-complaint relationships of various environmental odor sources in Styria, Austria

**DOI:** 10.1007/s11356-019-04410-z

**Published:** 2019-02-08

**Authors:** Lisbeth Weitensfelder, Hanns Moshammer, Dietmar Öttl, Ingrid Payer

**Affiliations:** 1 Department of Environmental Health, Center for Public Health, Medical University of Vienna, Kinderspitalgasse 15, 1090 Vienna, Austria; 2 Air Quality Control, Government of Styria, Landhausgasse 7, 8010 Graz, Austria

**Keywords:** Odor annoyance, Odor complaints, Odor dispersion modeling, Industrial odors, Agricultural odors, Logistic regression

## Abstract

In the planning and authorization process of industrial plants or agricultural buildings, it needs to be ensured that odor emissions do not annoy nearby residents in an unacceptable way. Previous studies have shown that odor-hour frequency is an important predictor for odor annoyance. However, odor-hour frequencies can be assessed for day and night separately. The present study relates complaint rates with different odor types and different metrics of frequency calculated via a dispersion model. Binary logistic regression analyses show that odor type and frequency of odor-hours are important predictors for complaints, while type of residential area does not increase the predictive value of the model. The combination of calculated frequency of day time odor-hours and type of odor explains complaint rates best. It is recommended to keep odor emissions as low as possible, especially for highly annoying odor types.

**Electronic supplementary material:**

The online version of this article (10.1007/s11356-019-04410-z) contains supplementary material, which is available to authorized users.

## Introduction

In the planning process of an industrial plant or an agricultural or any other building, it must be ensured that neither the construction work nor the intended use of the building cause emissions that endanger the health of the neighbors or lead to unacceptable annoyance. Since theoretically any perceived emission, even the color/design of a building’s façade, could cause annoyance in some individual, the respective Austrian laws (e.g., Republik Österreich 1994) state that the possibility to cause annoyance is not assessed on an individual case-by-case basis but rather assuming a hypothetical norm person. Furthermore, the Austrian laws do not stipulate that any annoyance is to be prevented, but only “unacceptable” annoyance—a term that needs to be interpreted in a legal context.

In the case of odorous emissions in the planning stage, emissions are usually estimated based on default assumptions, e.g., VDI 3894 (Verein Deutscher Ingenieure 2011), and a dispersion model (Oettl 2015; Oettl and Ferrero 2017; Janicke and Janicke 2007; Ritter et al. 2013) will provide information about the exposures at the neighbors’ places.

Some research tries to categorize the mentioned odor characteristics to five annoyance-predicting factors called FIDOL (Freeman and Cudmore 2002), namely frequency, intensity, duration, offensiveness, and location. While previous research often focuses on only some of these factors, suggestions have been made that all factors should be linked to obtain an expression for odor nuisance (Invernizzi et al. 2017), but a combination of all factors combined based on real data has not been made yet. Because of a high correlation between frequency, duration, and intensity in the case of a fairly continuous emission source, it is often not feasible to study the impact of all these factors together.

In Austria, odor exposure is usually assessed by measuring the frequency of perceived exposure defined as “odor-hours” (OH). An OH is defined as an hour during which odors exceed specific thresholds, usually one odor unit per m^3^ (OU) for more than 6 minute. Hence, the OH metric approach is based on short-term peaks in contrast to most other European countries which use an hourly mean (or percentile) approach for odor assessment. This OH approach that is typical for Austria and Germany reflects the characteristics of the human nose which is not very well adapted for measuring the concentration of an odorous substance. Humans can perceive a change in exposure but with continuous exposure, the sensory buds habituate or adapt quickly (Sirotin et al. 2015) rendering the assessment of absolute exposure practically impossible. Even regarding relative intensity, humans usually can only discern differences that are rather large when compared to visual discrimination thresholds (Barrett and Ganong 2010). Frequency of perception, usually defined as the percentage of hours during which a given threshold, often the threshold of perception, is exceeded in a defined number of minutes, can be estimated by dispersion models (Oettl and Ferrero 2017) and can be validated by field inspections (CEN 2017). Although perception thresholds differ between individuals, neighbors can at least hypothetically compare theses estimates and/or findings with their own observations. In another paper (Oettl et al. 2018), we report how the dispersion model used in this study performs in relation to the results of a field inspection (CEN 2017). In that demonstration case, the source emissions were measured through dynamic olfactometry (CEN 2003).

As odor emissions do not automatically result in annoyances of nearby residents, predicting and assessing annoyance is not a trivial task. Studies on odor annoyance usually used questionnaires addressed to the residents (Sucker et al. 2009; Aatamila et al. 2010; Boers et al. 2016; Miedema and Ham 1988; Miedema et al. 2000; Radon et al. 2004).

While a specific guideline (VDI 3883, part 1) especially focuses on annoyance assessment, both exposure assessment and questionnaire surveys are demanding and therefore still relatively scarce. While the process leading from odors to nuisance is already complex (van Harreveld 2001), complaints probably represent a rather severe annoyance because otherwise a citizen would not take the trouble to file a complaint. Complaining also depends on whether the utility of the complaint is considered high (Kowalski 1996), so positive effects (achieved goals, catharsis etc.) have to outweigh undesired consequences of the complaint.

The present study shows what can be done with data gained for administrative purposes, so several aspects leading to annoyance (and later to complaints) cannot be considered: apart from odor characteristics, personality aspects have been shown to be connected to annoyance, like dissatisfaction with health (Sucker et al. 2006; Steinheider and Winneke 1993). The importance of perceived health could also be shown in another study, where perceived pollution and health risk perception played important roles in predicting annoyance, possibly even more than exposure level (Claeson et al. 2013). Sucker et al. (2006) showed that also living quality and noise annoyance have an influence on the odor annoyance reaction of residents, even though odor frequency still remains a more important predictor. One predictor showed even more influence on the annoyance reaction than odor frequency, namely engagement in the neighborhood (Sucker et al. 2006): those residents who stated that they can imagine engaging for their neighbor’s interests also reported that they feel more annoyed by the same levels of odor nuisance. Correlations of odor annoyance with age are not reported consistently: some studies (Sucker et al. 2006; Steinheider et al. 1998) found a connection between age and annoyance, while others did not find demographic differences (Strauß and Cervinka 1987).

Furthermore, environmental conditions might represent an important parameter for annoyance. When comparing complaint statistics with time patterns of calculated odor sensations, there is a mismatch, showing that at specific times, odor is perceived more annoying (Schauberger et al. 2006). Complaints are more often in the warm season, which can be due to several minor reasons regarding perception or odor production, but mostly due to the time pattern of the behavior of residents who spend more time outside. Therefore, Schauberger et al. (2009) suggest that odor episodes should be weighted by the time of the day and the year regarding their annoyance potential.

However, personality-related contributors to annoyance cannot be considered, when it comes to administrative decisions, e.g., whether to expand an agricultural site or not. We do acknowledge that data collected for administrative purposes might be biased: dispersion models are either run routinely when a new source is planned or in the case of complaints in the case of an existing source. So, odor sources with complaints could be overrepresented. But also cross-sectional questionnaire surveys could be subject to participation bias. We acknowledge that frequency of complaints is not the same as percentage of severely annoyed persons. But we are confident that likelihood of complaints is by itself valuable information for the setting of limit values. “Highly” annoyed can have slightly different meanings depending on the language and the way the questions are phrased. Odor sources were operative since different dates and thus complaints were filed at different times and over different time periods. But because most complaints are filed in the first years of the operation of a new source, the different durations of operation do not strongly affect the number of complaints.

## Materials and methods

### Study aims

The following analysis attempts to predict residential odor complaints solely based on odor characteristics that can be calculated before constructing or expanding odor-emitting sites. Thereby default emission factors usually applied in Austria were used for agricultural odors (swine and fowl) and a dispersion model validated for use under Austrian geographical and meteorological conditions. The results shall give an overview on how far it is possible to predict percentage of odor complaints solely based on data available in the planning process and should inform evaluators on which frequency measure to use.

### Research methods, data collection, and sample

Utilizing data that were collected for administrative purposes over a period of 12 years, variables from different agricultural and industrial sites in Styria (Austria) are analyzed to find those parameters that predict complaints best. Sites were assessed during licensing procedures (e.g., for expansions) or in case of existing complaints. Odor frequency and intensity were calculated via dispersion modeling by the Graz Lagrangian Model GRAL (Oettl and Ferrero 2017). This dispersion model has been validated by field inspections for the purpose of odor modeling (Oettl et al. 2018). Odor frequencies as defined below were calculated for all residencies starting at the source and calculating exposure in every direction until zero odor hours were reached, i.e., the dispersion model indicated that at the façade of the residential building, the odor threshold (averaged over 6 min intervals) would never be exceeded. Annoyance was assessed via recorded complaints.

Odor frequencies are defined by the percentage of hours in which certain thresholds are exceeded in at least 10% of the time: %1OU describes the exceedance of one OU; so, a value of 20 means that in 20% of considered hours, an odor with a concentration of one OU is noticeable for at least 6 min per hour. One OU is the concentration that is perceived by 50% of all healthy people; %3OU represents three times the concentration. Percentages were calculated both for all 24 h of the day and for day time hours (6 a.m.–6 p.m., %1OU_Day). In another model, day- and night-time hours were weighted (%1OU_weighted) according to Schauberger et al. (2009). When model fit of the logistic regression was deemed unsatisfactory, the logarithmic values of %OU + 1 were used instead (log10 + 1). Level of significance is set to 0.05.

The following calculations include descriptive statistics and binary logistic regressions, whereas odor frequency (odor hours), odor quality (e.g., swine, fowl, animal bowel cleanse for sausage production), and residential area (open land, village area…) are considered as independent variables.

Overall, there are 392 data sets from 51 sites (50 operators) in Austria, representing an unbalanced sample favoring complaints of residential sites. Each data set represents either a complaint from a specific residential site or a data point from a residential site where no complaint was recorded. Table 1 shows odor qualities and complaints for the complete data set, while some variables (e.g., all types of odor hours) were not available for all sites.

**Table 1:** Complaints and odor quality in the data sample

		Swine	Fowl	Bowel cleanse	Biowaste	Sludge	Sludge and biowaste	Total
Complaints	no	110	8	9	104	48	19	298
	yes	36	5	4	17	32	0	94
Total		146	13	13	121	80	19	392

**Fig. 1:**
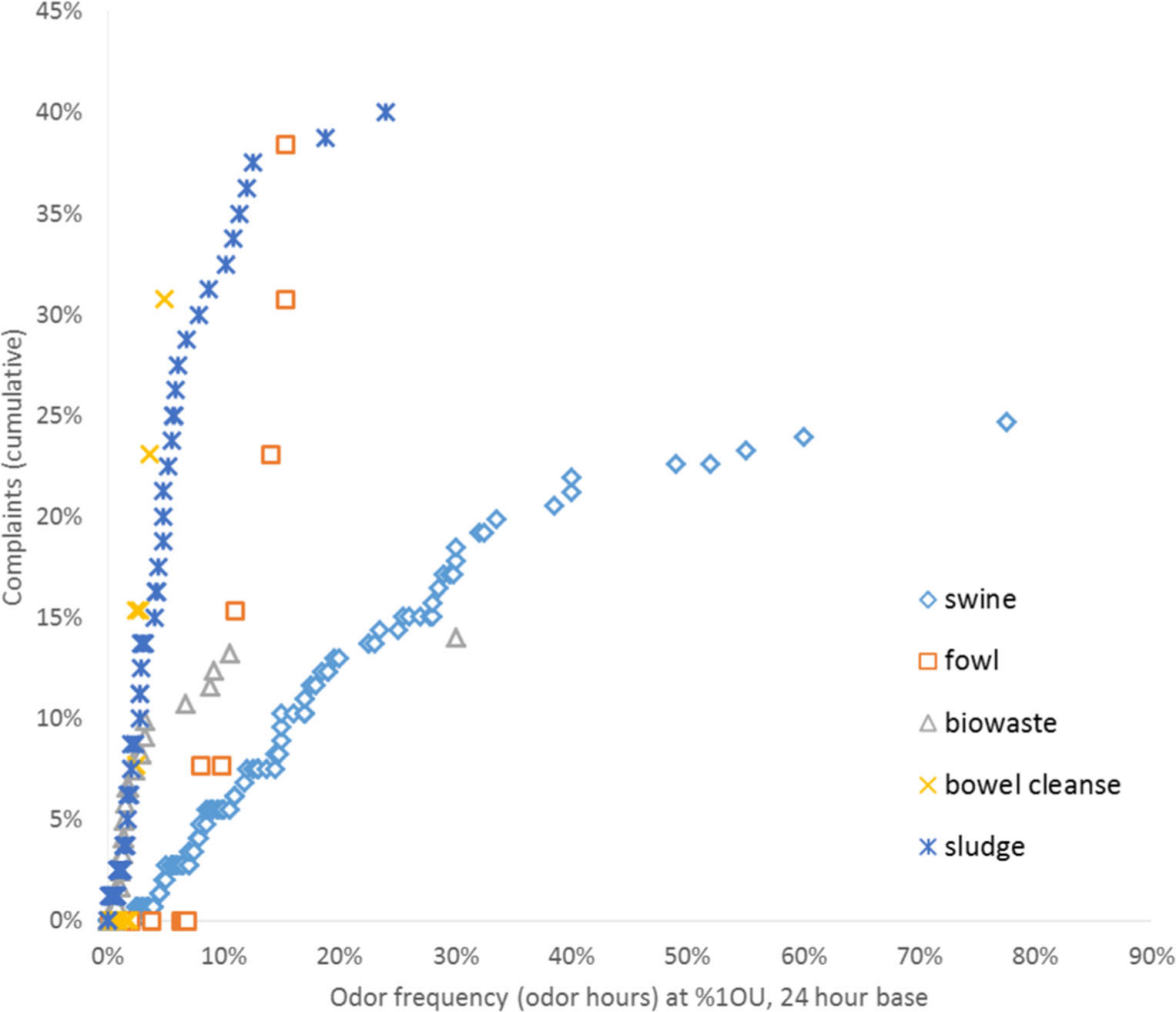
Cumulative complaint rates at different odor frequencies (24 h base, %1OU)

It seems plausible that the two types of compost works (biowaste, sludge) differ in their annoyance potential, and indeed, different complaint percentages were recorded (Pearson Chi-square = 17.592; *df* = 1, *p* < .001, *n* = 201), so they are considered separately in the subsequent analyses. The combination of sludge and biowaste (which did not cause any complaints, see Table 1) is removed due to missing information about the ratios of the two types (the removed 19 data points all belong to two different sites and operators). In spite of this removal of composting facilities with not well-defined input materials, the odorous substances emitted by the sites are clearly still very heterogeneous (Müller et al. 2004a, b). Figure 1 shows cumulative complaint rates for the remaining odor qualities at different odor frequencies (all with %1OU intensity on a 24 h base).

### Statistical analyses

In a first step, the various measures of exposure (%1OU, %3OU, %1OU_Day, and %1OU_weighted) were compared with each other by pairwise Pearson correlation. Because of their high correlation, only one exposure indicator at a time could be entered in the logistic regression models as explained in the second step:

Logistic regression models assessed complaints as dependent variable. Sites defined odor quality, and in the authorization process, most other individual site factors cannot be predicted. Therefore, clustering by sites was not examined. In the models, different measures of exposure were examined in raw and logarithmized format. As additional factors odor quality and type of residential area were tested. The models were compared based on their Nagelkerke’s *R*^2^ and through Hosmer Lemeshow statistics.

**Fig. 2:**
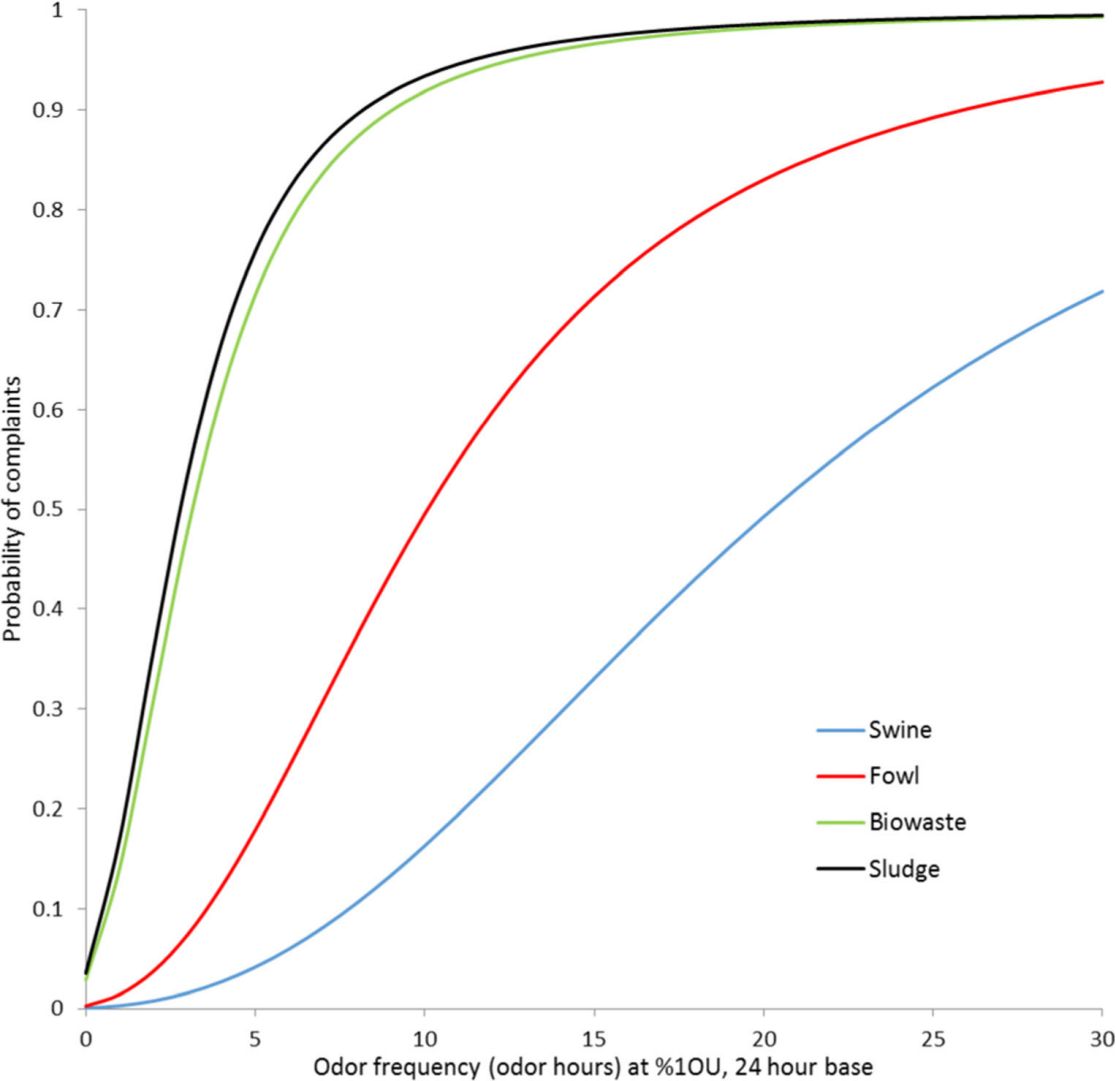
Results of the logistic regression (point estimates only) for different odor qualities, odor hours based on 24 h per day

To visualize the results of the logistic regression, the coefficients were entered into the logistic function, and the point estimates of the frequency-response curves for swine, fowl, biowaste, and sludge are presented in a figure (Fig. 2). To allow a comparison with other studies, %1OU (24 h base) was used as measure of exposure. For that figure, we calculated probability of complaint (Pr) for every OU applying the coeeficients of Table 2. For example, the curve for fowls was calculated usingthe following formula:

$$ \mathrm{Logit}\ \left(\Pr \right)=5.71{9}^{\ast}\left(\log \left(\mathrm{OU}\right)+1\right)+1.617\ \left(\mathrm{for}\ \mathrm{fowl}\right)\hbox{--} 7.592 $$

To calculate the probability of complaints (Pr), the logit function (logit) must be resolved:

$$ \Pr =\exp \left(\mathrm{logit}\right)/\left(\exp \left(\mathrm{logit}\right)+1\right) $$

**Table 2:** Summary of binary logistic regression analyses for different odor frequency measures

Variable	B	SE B	Exp(B)	*P*	Nagelkerke *R*^2^
Overall–log10, 1 OU^a^				< .001	.579
Frequency (day time)	6.949	.976	1041.901	< .001	
Quality (reference: swine)	0				
Quality: fowl	1.324	.785	3.759	.092	
Quality: bowel cleanse	4.248	1.063	69.971	< .001	
Quality: biowaste	4.399	1.014	81.358	< .001	
Quality: sludge	4.081	.807	59.195	< .001	
Constant	− 7.899	1.176	.000	< .001	
Overall–log10, 1 OU^b^				< .001	.487
Frequency (24 h base)	5.719	.700	304.647	< .001	
Quality (reference: swine)	0				
Quality: fowl	1.617	.717	5.040	.024	
Quality: bowel cleanse	4.295	.902	73.341	< .001	
Quality: biowaste	4.061	.755	58.058	< .001	
Quality: sludge	4.285	.641	72.613	< .001	
Constant	− 7.592	.898	.001	< .001	
Overall–log10, 1 OU^c^				< .001	.452
Frequency (weighted)	5.165	.648	175.091	< .001	
Quality (reference: swine)	0				
Quality: fowl	1.885	.712	6.589	.008	
Quality: bowel cleanse	3.212	.842	24.826	< .001	
Quality: biowaste	3.301	.688	27.142	< .001	
Quality: sludge	3.544	.563	34.611	< .001	
Constant	− 6.512	.783	.001	< .001	
Overall–raw data, 1 OU^d^				< .001	.484
Frequency (day time)	.375	.069	1.455	< .001	
Quality (reference: swine)	0				
Quality: fowl	1.988	.847	7.297	.019	
Quality: bowel cleanse	3.595	1.022	36.401	< .001	
Quality: biowaste	2.773	.907	16.004	.002	
Quality: sludge	3.606	.825	36.802	< .001	
Constant	− 5.029	.913	.007	< .001	
Overall–log10, 3 OU^e^				< .001	.513
Frequency (day time)	9.150	1.336	9411.015	< .001	
Quality (reference: swine)	0				
Quality: fowl	− 1.318	.925	.268	.154	
Quality: bowel cleanse	2.678	.937	14.549	.004	
Quality: biowaste	1.160	.653	3.190	.076	
Quality: sludge	1.766	.585	5.845	.003	
Constant	− 3.905	.671	.020	< .001	

*OU* odor unit/m^3^

^a^*n* = 302; Hosmer Lemeshow Chi-square: 9.589, *df* = 8, *p* = .295

^b^*n* = 373; Hosmer Lemeshow Chi-square: 13.380; *df* = 8; *p* = .099

^c^*n* = 373; Hosmer Lemeshow Chi-square: 3.699; *df* = 8; *p* = .883

^d^*n* = 302; Hosmer Lemeshow Chi-square: 25.161; *df* = 8; *p* = .001

^e^*n* = 302; Hosmer Lemeshow Chi-square: 6.727; *df* = 8; *p* = .566

## Results

The different indicators of odor exposure were highly correlated with each other. For %1OU, %3OU, and %1OU_Day, the pairwise correlation coefficients are between .85 (%3OU and %1OU_Day) and .93 (%1OU and %1OU_Day), all *p* < .001.

Figure 1 shows cumulative complaint rates for different odor qualities and frequencies (intensity: 1 OU, 24 h base). Fowl odors show a steep rise in complaint rates with increasing frequency, but the odors of sludge and bowel cleanse even exceed that annoyance potential. In an initial stepwise regression analysis, odor frequency (log 10 + 1) and odor quality are considered in a first step; adding type of residential area to the model in a subsequent step does not lead to a significant improvement compared to the model without it (Appendix Table 3), and also Nagelkerke *R*^2^ remains nearly the same.

Therefore, the subsequent analysis is done without type of residential area. Table 2 shows the results of the final binary logistic regression for different frequency data. For comparison, results are also calculated for unlogarithmized (raw) data and higher intensity (three OU instead of one OU). Figure 2 visualizes the results of the second model (%1OU, 24 h base).

The model with odor frequency measured at day time and with one OU shows the highest Nagelkerke *R*^2^. For day time frequency, different alternatives were analyzed, also showing why the use of logarithmized data can be necessary: when using unlogarithmized data, Nagelkerke *R*^2^ is lower (for frequency during day time: *R*^2^ = .484 instead of .579 when using logarithmized data), and the model assumptions are not met (significant Hosmer Lemeshow statistics). Also, the model with %1OU (logarithmized) seems to be a slightly better predictor than with %3OU (Table 2). Additional models considering %3OU and/or raw data variables can be found in Appendix Table 4) for comparison, with none of them explaining more variance than the model with %1OU day-time frequency as predictor.

## Discussion

### Annoyance and its connection to odor characteristics

Frequency of hours when 1 OU is exceeded is enough to predict annoyance, and considering a higher intensity does not increase the value to the model (Table 2). Even more, the model with just one OU seems to predict complaints better than the model with higher intensity, so it can be recommended for future site decisions to focus especially on the lower intensity.

When it comes to annoyance, previous studies suggest two findings regarding odor characteristics. First, while odor hours are a suitable predictor for annoyance, a German study concludes that odor intensity is not (Both et al. 2004). If an odor can be recognized, it can cause annoyance, even though some authors emphasize the necessity to disentangle odor frequency and odor intensity (Griffiths 2014). But results show that odor frequency (odor hours) is at least a sufficient annoyance predictor for neutral and unpleasant odors (Both et al. 2004), which leads to the second finding: the hedonic tone of odors plays a very important role. Several studies did not consider the hedonic tone, but it has a clear effect on the dose-response relationship whether odors are considered pleasant or unpleasant/neutral (Both et al. 2004; Sucker et al. 2008a) with a much lower annoyance potential of odors perceived as being pleasant.

It remains unclear whether different degrees of unpleasantness have an effect on annoyance. In the results of Both et al. (2004) resp. Sucker et al. (2008a) for both the neutral and unpleasant odors frequency is sufficient as a predictor for annoyance, resulting in a combined class of neutral and unpleasant odors (Sucker et al. 2008a; Sucker et al. 2008b), and even though the authors considered some of the odors “neutral”, data shows that they were rather considered less unpleasant than some other odor sources (Sucker et al. 2008b). The six odors used in the studies were diverse: sweets production and rusk bakery (pleasant), textile and seed oil production (neutral), fat refinery and cast iron foundry (unpleasant). Contrary to the mentioned results, an earlier study (Hangartner et al. 1998) suggested that there might be annoyance differences between “mildly” and “very” unpleasant odors, but for that last-mentioned study, different standards and unclear rating qualifications were used, so results are hard to compare. Our study results clearly support the notion that odor quality is a significant and relevant predictor of complaint rates. Therefore, for different odors, different frequencies should be deemed acceptable or unacceptable.

In a study that considered agricultural odors (Sucker et al. 2006), a negative relationship between intensity and hedonic tone was found for all odors which is characteristic of unpleasant odors (Sucker et al. 2008b). The authors noticed that it is important to differentiate between different types of animals, since cattle odors evoke less annoyance reactions than fowl or swine odors, though all investigated livestock odors were considered as unpleasant on the hedonic scale. However, they show different odor qualities when investigated with polarity profiles (rated via polar adjectives: Verein Deutscher Ingenieure 2010), where fowl odors show more similarities with a stench than swine odors. The results go along with an early study (Strauß and Cervinka 1987) where annoyance potential of fowl and swine odors is significantly higher than the annoyance due to cattle odors. Though not reporting for significance, Sucker (2010) listed cattle odors as the odors with the least annoyance potential and fowl odors to be the ones with the highest annoyance potential. The different agricultural odor qualities are also weighted differently in the guideline for odor impact in Germany (GOAA: North Rhine-Westphalia 2008), with the highest weighting factor for fowl and the lowest for cattle. This is also in line with our study results where odors from fowls led to more frequent complaints than odors from swine. Although cattle husbandry is also prevalent in Styria, no complaints have been filed so far about cattle odor in that country.

Apart from odor frequency, odor quality is important for predicting complaint rates, but type of residential area is not. With our regression model, nearly 58% of the variance in the likelihood of complaints can be explained by the variables odor quality and odor frequency during day time.

The fact that neither type of residential area (as resemblance to location) nor a higher odor intensity add value to the regression model does not affirm researchers who emphasize the use of all five FIDOL characteristics, and less predictors can already be seen as sufficient.

It is worth mentioning that in the present sample, the model with odor frequency evaluated for the day time explained complaint rates best. Also in the weighting model of Schauberger et al. (2009), odor sensations during day time is considered more important due to the residents’ typical daily behavior patterns. In the present sample, an unweighted model considering only day time odor frequency fits the data even better than the weighted model. This has an important practical consequence for exposure assessment, where according to the given results, it would be sufficient to solely assess odor frequency during day time. Nevertheless, it needs careful consideration, whether odor frequency during night time should be ignored, since research about health consequences of nightly odor emissions is scarce.

### Study limitations

Limiting the given results, it has to be mentioned that sample size was rather small for some odor qualities. In addition, the given convenience sample is probably not representative for agricultural sites in general, since the majority of sites included in this study are those, where complaints have been actively reported to the local administrations. Several control variables (e.g., personality) were not available, which could still have led to an even better explanation of complaint behavior. Instead, the research contains “hard” site facts that are available when decisions for site expansions or site constructions are to be made, so that it is especially of practical value and should stimulate future research. Also, complaint rates are hard to compare with results from previous studies based on questionnaires, since different nuances of annoyance cannot be deduced. However, it is likely that filing a complaint already represents a severe annoyance. To support that assumption, research on complaint vs. annoyance rates shall be encouraged, since usually research about dose-response-relationships (e.g., Miedema and Ham 1988; Miedema et al. 2000) focuses on annoyance.

As a final limitation, it has to be mentioned that odor frequency was calculated based on assumptions that were made before sites were actually constructed or expanded, so it is possible that these assumptions might have over- or underestimated the real situation. A recent study found evidence that the German factor-4 model rather overestimates the odor impact in general, while odor hours computed with the recently developed concentration-variance model, as it was done in the present study, agree rather well with odor hours obtained by field inspections (Oettl et al. 2018). Nevertheless, dispersion calculations can only be as good as the emission assumptions they are based on. Important factors determining the level of odor emissions (e.g., animal feeding techniques) might underlie a rapid change, so emission factors listed in guidelines, like the VDI 3894 (Verein Deutscher Ingenieure 2011) used in this work, should be evaluated constantly. It remains a task for ongoing research, to improve emission factors. It needs to be stressed that a bias in emission factors would also result in an absolute bias in the dose-response relationships presented in this work.

## Conclusions

Odor frequency assessed during day time exceeding one OU is the best predictor for complaints. While odor quality also serves as a predictor for complaints, no additional predictive value of type of residential area could be found. Especially for highly annoying odors (bowel cleanse, sludge, fowl), odor emissions should be kept as low as possible. For these odors even with a frequency of only 2% (%1OU and %1OU_Day), there is a high probability of annoyance and complaints.

The paper also demonstrates that the use of data produced for administrative purposes is possible and can generate relevant information. More frequent use of similar data sets and a stronger cooperation between public health administration and academia are encouraged.

### Electronic supplementary material

ESM 1 (XLSX 44 kb)

## Appendix

The following appendix contains an additional block-wise calculation for the regression analysis (%1OU, day time; Appendix Table 3) as well as additional binary logistic regression analyses for comparison (Appendix Table 4).

**Table 3:** Chi-squares and model summary for the stepwise (blockwise) analysis

Variables	*χ*^2^ model (*χ*^*2*^ block)	Nagelkerke *R*^2^	*p* model (*p* block)
Block 1: Odor frequency^a^ and quality	146.910 (146.910)	.568	< .001 (< .001)
Block 2: Type of residential area^b^ added	147.330 (.420)	.569	< .001 (.811)

*n* = 298

^a^Frequency values for day time and 1 OU (odor unit/m^3^)

^b^Type of residential area includes open land, village, and urban residential district; industry area was not considered due to the low number of data points (*n* = 4)

Regarding additional logistic regression analyses, it can be seen that the model does not represent the data well when raw data is used on a 24 h based frequency (significant Hosmer Lemeshow statistics; Appendix Table 4). The model with day time frequency results in a higher Nagelkerke *R*^2^ than the models with frequency calculated on a 24 h base.

**Table 4:** Summary of additional binary logistic regression analyses for data with %3OU and unlogarithmized data

Variable	B	SE B	Exp(B)	*p*	Nagelkerke *R*^2^
Overall–log10, 3 OU^a^				< .001	.332
Frequency (24 h base)	4.277	.607	72.037	< .001	
Quality (reference: swine)	0				
Quality: fowl	1.125	.707	3.079	.112	
Quality: bowel cleanse	2.447	.757	11.552	.001	
Quality: biowaste	1.197	.482	3.309	.013	
Quality: sludge	2.288	.443	9.851	< .001	
Constant	− 3.515	.464	.030	< .001	
Overall–raw data, 1 OU^b^				< .001	.317
Frequency (24 h base)	.152	.024	1.164	< .001	
Quality (reference: swine)	0				
Quality: fowl	1.587	.671	4.889	.018	
Quality: bowel cleanse	2.299	.734	9.964	.002	
Quality: biowaste	1.351	.504	3.860	.007	
Quality: sludge	2.490	.461	12.060	< .001	
Constant	− 3.386	.454	.034	< .001	
Overall–raw data, 3 OU^c^				< .001	.468
Frequency (day time)	1.574	.277	4.852	< .001	
Quality (reference: swine)	0				
Quality: fowl	− 1.291	1.070	.275	.228	
Quality: bowel cleanse	2.017	.852	7.516	.018	
Quality: biowaste	.833	.589	2.301	.157	
Quality: sludge	1.785	.541	5.961	.001	
Constant	− 3.153	.555	.043	< .001	
Overall–raw data, 3 OU^d^				< .001	.212
Frequency (24 h base)	.248	.050	1.282	< .001	
Quality (reference: swine)	0				
Quality: fowl	1.067	.633	2.907	.092	
Quality: bowel cleanse	1.241	.667	3.460	.063	
Quality: biowaste	.202	.384	1.224	.598	
Quality: sludge	1.523	.357	4.588	< .001	
Constant	− 2.101	.294	.122	< .001	

*OU* odor unit/m^3^

^a^*n* = 371; Hosmer Lemeshow Chi-square: 7.638, *df* = 8, *p* = .470

^b^*n* = 373; Hosmer Lemeshow Chi-square: 35.071, *df* = 8, *p* < .001

^c^*n* = 373; Hosmer Lemeshow Chi-square: 9.319, *df* = 8, *p*.316

^d^*n* = 371; Hosmer Lemeshow Chi-square: 26.716, *df* = 8, *p* = .001
